# Biopolymer Nanofibers for Nanogenerator Development

**DOI:** 10.34133/2021/1843061

**Published:** 2021-02-22

**Authors:** Lulu Bai, Qing Li, Ya Yang, Shengjie Ling, Haipeng Yu, Shouxin Liu, Jian Li, Wenshuai Chen

**Affiliations:** ^1^Key Laboratory of Bio-Based Material Science and Technology, Ministry of Education, Northeast Forestry University, Harbin 150040, China; ^2^CAS Center for Excellence in Nanoscience, Beijing Key Laboratory of Micro-Nano Energy and Sensor, Beijing Institute of Nanoenergy and Nanosystems, Chinese Academy of Sciences, Beijing 101400, China; ^3^School of Physical Science and Technology, ShanghaiTech University, Shanghai 201210, China

## Abstract

The development of nanogenerators (NGs) with optimal performances and functionalities requires more novel materials. Over the past decade, biopolymer nanofibers (BPNFs) have become critical sustainable building blocks in energy-related fields because they have distinctive nanostructures and properties and can be obtained from abundant and renewable resources. This review summarizes recent advances in the use of BPNFs for NG development. We will begin by introducing various strategies for fabricating BPNFs with diverse structures and performances. Then, we will systematically present the utilization of polysaccharide and protein nanofibers for NGs. We will mainly focus on the use of BPNFs to generate bulk materials with tailored structures and properties for assembling of triboelectric and piezoelectric NGs. The use of BPNFs to construct NGs for the generation of electricity from moisture and osmosis is also discussed. Finally, we illustrate our personal perspectives on several issues that require special attention with regard to future developments in this active field.

## 1. Introduction

With the fast development of modern society, the question of how to ease the increasing energy demand in various fields has become one of the most critical issues facing human beings [1–4]. Although numerous mature systems for energy storage, conversion, and harvesting have been established, many potential sources of energy in people's daily lives—especially in disordered forms such as human movement and wind energy—are still largely wasted. Therefore, the development of advanced materials and systems for harvesting various types of energy is critical for power generation. Compared with other strategies for electricity generation, the use of nanogenerators (NGs) has some obvious advantages. These include high power output, flexible structural design and assembly, diverse material selection, and the ability to transform disordered forms of energy into electricity [5–9]. Piezoelectric NGs (PENGs) [10], triboelectric NGs (TENGs) [11], pyroelectric NGs [12], and hybrid NGs [13] represent an emerging branch of energy conversion and have been widely developed. TENGs harvest mechanical energy by the coupling effect of contact electrification and electrostatic induction [14]. When using TENGs, two different materials periodically contact each other and separate. This results in the flow of induced electrons between two electrodes. TENGs are mainly divided into vertical contact-separation, lateral-sliding, single-electrode, and free-standing working modes [15]. PENGs depend on the structural particularity of piezoelectric materials, which are crystalline substances with noncentrosymmetry. During external mechanical stimulation, the lattice dipole moment in the piezoelectric material deforms, causing polarization. This results in the generation of an external potential difference [16]. Various polymers such as fluorinated ethylene propylene (FEP) [17], polyethylene terephthalate (PET) [18], polytetrafluoroethylene (PTFE) [19, 20], polydimethylsiloxane (PDMS) [21], polyimide (PI) [22], and polyvinyl chloride (PVC) [23] have been widely researched in the designing of TENGs, ranging from material selection and modification, structure design, and patterning to performance optimization and applications [24]. PENGs have been developed from various piezoelectric materials such as zinc oxide (ZnO) nanowires [10], cadmium sulfide (CdS) nanowires [25], poly(vinylidene fluoride) (PVDF) [26], and barium titanate (BTO) nanoparticles [27]. Various polymers such as PDMS [28, 29] have been integrated with piezoelectric materials to fabricate PENGs with improved mechanical strength and flexibility. However, the above-mentioned polymers are mainly synthetic polymers at relatively high cost, and their fabrication is generally complicated or requires toxic solvents. Therefore, there is an expectation that sustainable polymers, which could also provide novel building blocks for optimizing NG performance, will be exploited. Among the numerous candidates, biopolymer nanofibers (BPNFs) have attracted increasing interest as critical components for NGs.

BPNFs are natural polymer nanofibers that are synthesized by living things such as trees, bamboo, crabs, shrimps, spiders, and silkworms [30, 31]. Polysaccharide and protein nanofibers are the two most prominent types of BPNFs on earth. Polysaccharide nanofibers—such as cellulose [32–40] and chitin nanofibers [41]—generally have complex structural patterns and are used to support the bodies and guarantee the survival of biological organisms. Some protein nanofibers—such as collagen and keratin nanofibers—exist within biological organisms [42], whereas others such as silk nanofibers are secreted by silkworms or spiders [43–45]. Exfoliation is an effective strategy for individualizing BPNFs that inherit their original structures from biological materials [30]. Moreover, bacterial cellulose (BC), which is a special kind of polysaccharide nanofiber, can be obtained by biosynthesis [46–48]. Electrospinning is another versatile strategy for producing BPNFs from a viscoelastic biopolymer solution under a strong electric field [49, 50]. Different fabrication strategies and raw materials generate BPNFs with various structures and performances, providing ample building blocks for NGs.

Owing to their intrinsic nanostructures and properties, BPNFs have attracted increasing interest and have been widely utilized in material, energy, environmental, and biomedical fields [30]. The use of BPNFs in NG research has attracted tremendous attention. Most researchers have focused on the development of BPNF-based TENGs [51, 52] or PENGs [53, 54], but there has also been progress in the use of BPNF-derived generators for harvesting electricity from moisture [55] and osmosis [56]. Based on the nanofiber structures and properties, modification, integration, bulk material preparation, device assembly, and applications, the utilization of BPNFs for NGs is mainly categorized into the following five groups:

1. Owing to their intrinsic one-dimensional (1D) nanofiber structures and advantageous mechanical properties, BPNFs can be directly assembled or integrated with active materials to produce high-performance films, mats, membranes, or aerogels for various types of NGs
2. The surfaces of BPNFs are rich in active groups—such as hydroxyl, acetamide, amino, or carboxyl groups—that are recognizable active sites for chemical modification to modulate the properties of the BPNFs and BPNF-derived bulk materials for NGs
3. Owing to the intrinsic structures of BPNFs, NGs with special advantages such as flexibility, foldability, and transparency can be constructed by utilizing optimized BPNFs as building blocks and choosing appropriate methods for their assembly as bulk materials
4. Because of the easily processing of BPNFs into bulk materials, it is convenient for manipulating the characters of bulk materials, including their pore size, porosity, roughness, nanofiber alignment, patterning, and ability to form assemblies with complex architectures, to further improve the output performance of NGs
5. BPNFs with various widths, lengths, bundles, components, surface chemistries, and mechanical/thermal/optical properties can be produced from numerous sources using different fabrication strategies, providing abundant building blocks for the construction of many types of BPNF-derived NGs

There is increasing interest in utilizing BPNFs to construct NGs. Sometimes this simply involves using the BPNFs as building blocks to fabricate bulk materials as parts of the NGs. Sometimes these utilizations are built upon the intrinsic structures and tunable structures, components, functional groups, and properties of BPNFs and BPNF-derived bulk materials, which enable the generation of NGs with unique functions and high output performances. Owing to the efforts of many research groups, numerous BPNFs have been used to fabricate various types of NGs. Several reviews have focused on various aspects of the use of biopolymers to construct NGs [24, 57–61]. Some of them have summarized the utilization of several types of BPNFs such as nanocellulose for NG development. However, to date, there has not been a review that systemically summarizes and compares the utilization of various kinds of BPNFs for multiple types of NGs.

**Figure 1:**
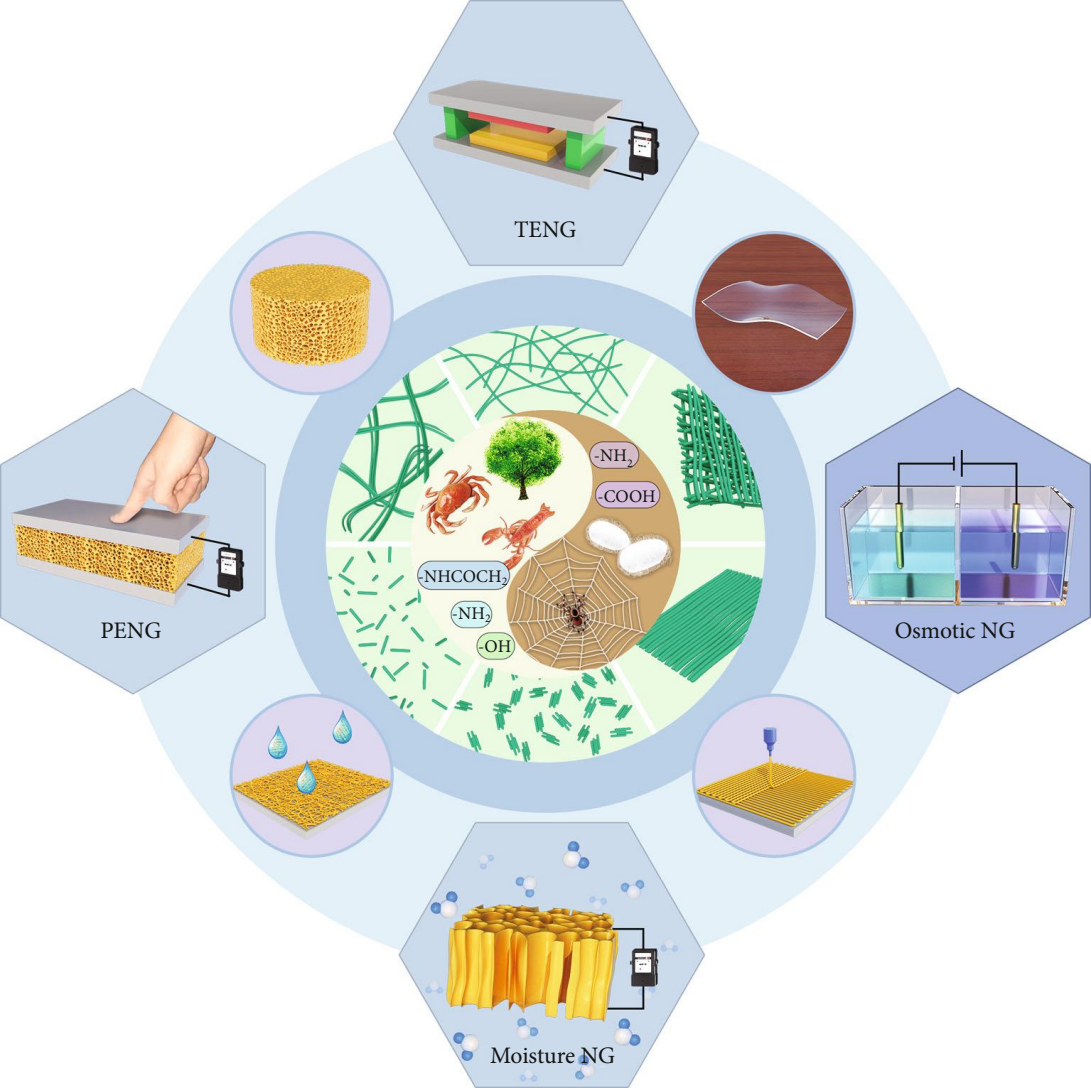
Schematic diagram showing the main topics of the present review, ranging from the sources, fabrication, and structures of biopolymer nanofibers to the construction of bulk materials and the use of bulk materials for the development of various nanogenerators.

Herein, we attempt to systematically summarize the generation of BPNFs with different structures, components, and performances for the development of NGs (Figure 1). We begin with an overview of various strategies that have been used to fabricate BPNFs from different sources and the structures and characteristics of various kinds of BPNFs. Then, we systematically summarize recent progress in the utilization of BPNFs for NGs. We will mainly focus on the development of BPNF-based TENGs and PENGs. We will also discuss recent advances in the use of BPNFs for harvesting electricity from moisture and osmosis. Finally, we provide a brief summary, together with personal perspectives on the challenges facing future research.

## 2. Fabrication of BPNFs

High-performance NGs require BPNFs with controlled structures and properties. Therefore, effective strategies for fabricating such materials are expected. To date, numerous methods for the generation of BPNFs have been established and optimized. These approaches fall into one of three categories: (1) exfoliation from biological materials, (2) biosynthesis from small molecules, and (3) electrospinning. Although nanofibers can also be obtained from biopolymers through dissolving-regeneration processes [62–66], they generally exist in films or aerogels; they have not been extracted as single individual nanofibers and are therefore not discussed in depth in the present review [67, 68]. Fabrication from different sources via various methods produces many types of BPNFs with different morphologies, lengths, widths, degrees of nanofibrillation, bundles, crystallinities, zeta potentials, chemical components, and surface chemistries. This section mainly comprises a discussion of the strategies used for BPNF fabrication.

### 2.1. Exfoliation

In biological materials, BPNFs mainly exist as polysaccharide or protein nanofibers and assemble into sophisticated hierarchical architectures with multistage scales. Exfoliation is an efficient “top down” strategy for individualizing these nanofibers. Because nanofibers are embedded in various matrices within most biological materials, chemical pretreatment is generally carried out before nanofibrillation. The structures and performances of different nanofibers and biological materials differ. Therefore, exfoliation strategies for various polysaccharide and protein nanofibers also generally differ, although there are some similarities. BPNFs with various structures and properties can be produced from any native biopolymer using different exfoliation strategies. The resulting BPNFs are quite slender. However, the large-scale extraction of high-quality nanofibers that are uniform in size and have a high degree of nanofibrillation using an environmentally friendly method remains challenging.

#### 2.1.1. Polysaccharide Nanofibers

**Figure 2:**
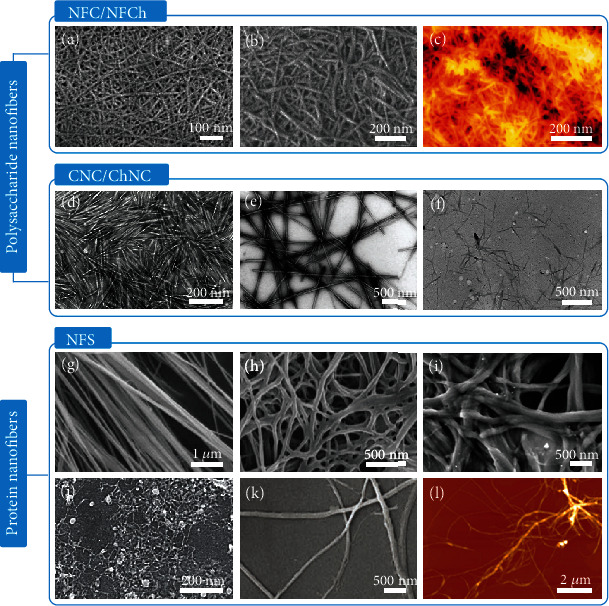
Fabrication of biopolymer nanofibers by exfoliation. SEM images of (a) nanofibrillated cellulose (NFC) and (b) nanofibrillated chitin (NFCh). (c) AFM image of TEMPO-oxidized NFC. TEM images of (d) cotton cellulose nanocrystal (CNC), (e) tunicate CNC, and (f) chitin nanocrystal (ChNC). SEM images of nanofibrillated silk (NFS) derived from (g) spider silk and (h) silkworm silk fabricated by high-intensity ultrasonication. (i) SEM image of NFS produced by milling combined with homogenization. SEM images of NFS fabricated by partially dissolving the degummed silk in (j) HFIP and (k) sodium hypochlorite before ultrasonication. (l) AFM image of NFS prepared by treating the degummed silk with HFIP for 72 h. (a) is reproduced with permission from Ref. [74], copyright 2007 *Biomacromolecules*. (b) is reproduced with permission from Ref. [76], copyright 2009 *Biomacromolecules*. (c) is reproduced with permission from Ref. [82], copyright 2009 *Biomacromolecules*. (d) is reproduced with permission from Ref. [71], copyright 2014 *ChemSusChem*. (e) is reproduced with permission from Ref. [86], copyright 2008 *Biomacromolecules*. (f) is reproduced with permission from Ref. [88], copyright 2007 *Biomacromolecules*. (g) and (h) are reproduced with permission from Ref. [91], copyright 2007 *Applied Physics Letters*. (i) is reproduced with permission from Ref. [92], copyright 2019 *ACS Sustainable Chemistry & Engineering*. (j) is reproduced with permission from Ref. [93], copyright 2016 *Advanced Materials*. (k) is reproduced with permission from Ref. [94], copyright 2018 *Advanced Functional Materials*. (l) is reproduced with permission from Ref. [99], copyright 2020 *ACS Materials Letters*.

Polysaccharide nanofibers mainly comprise cellulose nanofibers and chitin nanofibers. Chemically, cellulose is a linear long-chain polymer composed of *β*-1,4-linked anhydro-D-glucose units [69], whereas chitin is a cellulose analogue consisting of *β*-1,4-linked *N*-acetyl glycosaminoglycan repeating units [41]. Cellulose nanofibers exist in plants (such as trees and bamboo), in crop straws, and in some animals (such as tunicates) [32, 34, 35, 37, 38]. Chitin nanofibers mainly occur in the exoskeletons of shellfish and insects and in the cell walls of mushrooms [41]. In these biological materials, the polysaccharide nanofibers are mainly embedded in various matrices. For example, cellulose nanofibers usually form hybrid structures with hemicellulose and lignin within wood, whereas chitin nanofibers interact with proteins and calcium carbonate within crab shells. To facilitate the nanofibrillation and fabrication of high-purity cellulose and chitin nanofibers, the matrix should be largely removed in advance. Lignin can be removed by various chemical methods such as treatment with acidified sodium chlorite [70–72]. Hemicellulose can be largely eliminated by treatment with potassium hydroxide [70, 72–74]. Proteins and minerals can be removed using aqueous sodium hydroxide/potassium hydroxide and hydrochloric acid, respectively [75–77]. The as-generated purified cellulose and chitin are then subjected to nanofibrillation using various equipment such as high-pressure homogenizer [78, 79], grinder [74, 76], high-intensity ultrasonicator [70, 72], and high-speed blender [80, 81], resulting in the fabrication of high aspect ratio nanofibrillated cellulose (NFC) and nanofibrillated chitin (NFCh). NFC and NFCh are entangled and display web-like structures (Figures 2(a) and 2(b)) [74, 76]. Owing to the strong interactions through hydrogen bond and van der Waals forces among the adjacent nanofibers of biological materials, NFC and NFCh also contain nanofiber bundles. Electrostatic repulsion has been explored as a means of further improving the degree of nanofibrillation and facilitating the exfoliation of single nanofibers. The chemical modification of cellulose pulp by 2,2,6,6-tetramethylpiperidine-1-oxyl radical- (TEMPO-) mediated oxidation causes the selective generation of numerous C_6_ carboxylate groups on the nanofiber surfaces [82–84]. Electrostatic repulsion and/or osmotic effects lead to the exfoliation of individualized NFC with widths of 3–4 nm through mechanical nanofibrillation (Figure 2(c)) [82]. To isolate chitin nanofibers, various organic acids such as acetic acid are used for cationization of the C_2_ amino groups of the chitin [76, 85]. The electrostatic repulsions between the nanofibers promote the facile nanofibrillation and exfoliation of highly individualized NFCh.

Polysaccharide nanofibers can also be produced using a strong acid hydrolysis method to remove the amorphous area, leading to the generation of polysaccharide nanocrystals that are resistant to attack by strong acids. Compared with NFC and NFCh, the polysaccharide nanocrystals are short and highly crystalline. Cellulose nanocrystal (CNC) can be isolated by hydrolyzing cellulose pulp using various strong acids such as sulfuric acid (Figures 2(d) and 2(e)) [71, 86], hydrochloric acid [71], and phosphoric acid [87]. Chitin nanocrystal (ChNC) is generally extracted by hydrochloric acid hydrolysis (Figure 2(f)) [88], although sulfuric or phosphoric acid hydrolysis, TEMPO-mediated oxidation [89], and surface cationization [90] are also effective. The exfoliation of polysaccharide nanocrystals is widely determined by the resources, the type of acid used, the acid concentration, the polysaccharide/acid ratio, the hydrolysis temperature and duration, and the subsequent mechanical treatment [30, 33]. Therefore, different types of polysaccharide nanocrystals have apparently different structures and performances.

#### 2.1.2. Protein Nanofibers

Protein nanofibers are mainly found in protein-based biological materials such as silkworm and spider silk. A protein consists of one or more linear chains of amino acids. It is still a challenge to retain the native silk nanofiber structure during the exfoliation process. Nanofibrillated silk (NFS) can be prepared by directly nanofibrillating spider or silkworm silk or degummed silkworm silk by high-intensity ultrasonication (Figures 2(g) and 2(h)) [91] or milling combined with homogenization treatment (Figure 2(i)) [92]. The as-produced NFS still contains numerous bundles. The degree of nanofibrillation is increased by partial dissolution before the nanofibrillation process. Hexafluoroisopropanol (HFIP) is used to partially dissolve the degummed silk fibers. This has a critical effect on the subsequent ultrasonication process. The as-fabricated NFS is 20 ± 5 nm wide and up to 500 nm long (Figure 2(j)) [93]. HFIP can be replaced with nontoxic sodium hypochlorite (Figure 2(k)) [94]. The structure of NFS can be manipulated by controlling the input of the sonification energy. Ribbon-like NFS (~0.4 nm thick) can be produced by partially dissolving the degummed silk in a sodium hydroxide/urea solution [95] or subjecting it to TEMPO-mediated oxidation [96], in combination with ultrasonication treatment. NFS can also be extracted using various solvent systems such as salt/formic acid and HFIP without an additional mechanical process [97, 98]. When degummed silkworm silk fibers are treated with HFIP, individualized NFS with high aspect ratios, widths of approximately 3.1 nm, and perfectly preserved pristine conformations can be gradually exfoliated by controlling the duration of incubation (Figure 2(l)) [99].

### 2.2. Biosynthesis

In addition to exfoliation from native biological materials, another efficient strategy for the fabrication of high crystalline BPNFs is the biosynthesis of BC by the fermentation of microorganisms [46–48]. Previously, BC has mainly been produced for a popular dessert known as Nata de Coco. It was first reported by Brown in 1886 [100]. Low molecular weight carbon sources such as d-glucose are used as precursors for the synthesis of BC. Compared with cellulose nanofibers exfoliated from higher plants, BC nanofibers comprise pure cellulose, which can be biosynthesized on a large scale. However, BC ribbons are a little wide and thick. BC is generally formed into pellicles and is not well individualized. Moreover, BC is relatively expensive to produce.

**Figure 3:**
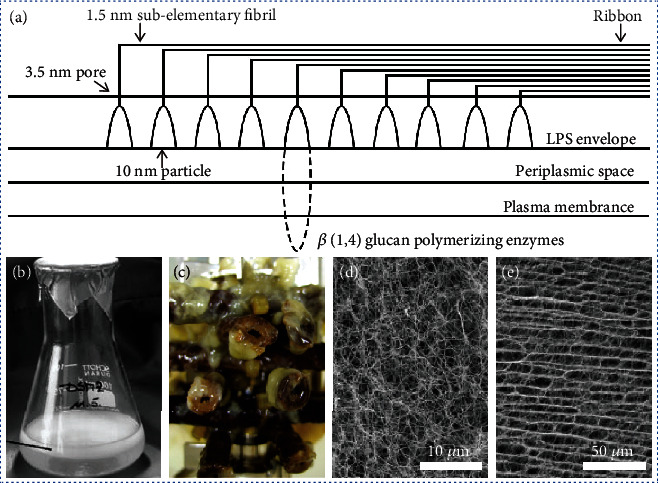
Fabrication of bacterial cellulose (BC) by biosynthesis. (a) Schematic showing the fabrication of BC. Digital images of BC fabricated under (b) static and (c) agitated conditions. SEM images of the (d) surface structures and (e) vertical section structures of BC pellicles. (a) is reproduced with permission from Ref. [101], copyright 1998 *Polymer Degradation and Stability*. (b) is reproduced with permission from Ref. [104], copyright 2006 *Polysaccharides II*. (c) is reproduced with permission from Ref. [105], copyright 2011 *Biomacromolecules*. (d) and (e) are reproduced with permission from Ref. [108], copyright 2008 *Advanced Materials*.

Several bacteria have been developed for the extracellular secretion of BC [101]. *Acetobacter xylinum* is the most efficient synthesizer of BC. In *Acetobacter xylinum*, BC is formed between the outer and cytoplasma membranes by a cellulose-synthesizing complex [102, 103] that is associated with the pores at the surface of the bacterium (Figure 3(a)) [101]. Large amounts of multienzyme complex systems participate in the synthesis, and the synthesis is a multistep reaction process. Following a complex synthesis, BC is assembled and excreted by the bacteria. It can be synthesized in both static (Figure 3(b)) [104] and agitated (Figure 3(c)) [105] conditions [106]. The structure of the BC can be controlled by manipulating various parameters such as pH, temperature, and incubation time during the fabrication process [46, 107].

The as-produced BC comprises ribbon-like nanofiber structures with high aspect ratios (Figures 3(d) and 3(e)) [108]. The ribbons are 20–100 nm wide, approximately 10 nm thick, and several micrometers long and are organized by ultrafine cellulose nanofibers. BC forms as pellicles with web-like entangled networks containing large amounts of water. It is pure cellulose, without the impurities that commonly exist in plant resources, such as hemicellulose and lignin. Generally, BC has a crystallinity of 70–80% and a degree of polymerization up to 8000 [109]. In recent years, BC has frequently been used for developing functional materials/devices in biomedical-, environment-, and energy-related fields. BC can be modified by introducing active components such as functionalized glucose [110] and carbon nanotubes [111] during microbial fermentation to produce a series of novel materials.

### 2.3. Electrospinning

Electrospinning is a facile, versatile, and efficient strategy for fabricating nanofibers from biopolymers. The electrospinning device comprises four main parts: a high-voltage power supply, a syringe pump, a spinneret, and a conductive collector [112–114]. Many biopolymers—including cellulose, chitin, chitosan, silk, collagen, and gelatin—can be electrospun into nanofibers, as long as they can be dissolved in appropriate solvents to obtain solutions that meet the requirements of electrospinning. Electrospun nanofibers are long and continuous and have uniform widths, although their widths are generally larger than those of exfoliated nanofibers. The native crystalline structures are usually destroyed when the biopolymers are dissolved. The critical challenge is to design and develop versatile, environmentally friendly, and inexpensive biopolymer solvents for electrospinning.

**Figure 4:**
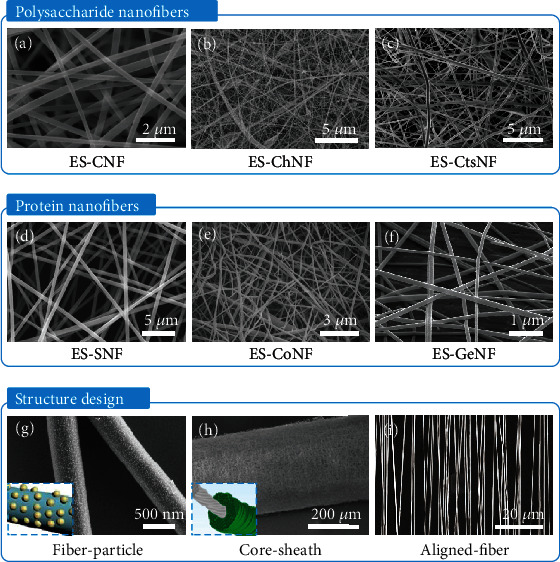
Fabrication of biopolymer nanofibers by electrospinning. SEM images of (a) electrospun cellulose nanofibers (ES-CNFs), (b) electrospun chitin nanofibers (ES-ChNFs), (c) electrospun chitosan nanofibers (ES-CtsNFs), (d) electrospun silk nanofibers (ES-SNFs), (e) electrospun collagen nanofibers (ES-CoNFs), (f) electrospun gelatin nanofibers (ES-GeNFs), (g) ES-SNF/gold nanoparticle composite fibers, (h) carbon nanotube yarn/ES-SNF core-sheath composite fibers, and (i) aligned ES-SNFs. (a) is reproduced with permission from Ref. [115], copyright 2020 *ACS Nano*. (b) is reproduced with permission from Ref. [116], copyright 2004 *Polymer*. (c) is reproduced with permission from Ref. [117], copyright 2007 *Biomacromolecules*. (d) is reproduced with permission from Ref. [118], copyright 2016 *Nano Research*. (e) is reproduced with permission from Ref. [119], copyright 2004 *Frontiers in Bioscience-Landmark*. (f) is reproduced with permission from Ref. [120], copyright 2013 *Advanced Functional Materials*. (g) is reproduced with permission from Ref. [166], copyright 2012 *Nano Letters*. (h) is reproduced with permission from Ref. [167], copyright 2018 *Nano Letters*. (i) is reproduced with permission from Ref. [168], copyright 2011 *Advanced Functional Materials*.

The solvent plays an important role with regard to the biopolymer in electrospinning. It should dissolve the crystalline biopolymer to generate an electrified jet. The solvent molecules should also be removed from the nanofibers by rapid vaporization after the nanofibers have been collected. Owing to the diversity and complexity of biological materials, particular solvents are required to fabricate electrospun cellulose nanofibers (ES-CNFs), electrospun chitin nanofibers (ES-ChNFs), electrospun chitosan nanofibers (ES-CtsNFs), electrospun silk nanofibers (ES-SNFs), electrospun collagen nanofibers (ES-CoNFs), and electrospun gelatin nanofibers (ES-GeNFs) (Figures 4(a)–4(f)) [115–120]. Because of the high crystallinity caused by the strong inter- and intramolecular hydrogen bonds, polysaccharides such as cellulose and chitin are generally difficult to dissolve. The commonly used solvents for electrospinning cellulose include *N*-methylmorpholine *N*-oxide (NMMO)/H_2_O [121–123], lithium chloride/*N*,*N*-dimethylacetamide (DMAc) [124–126], ionic liquid (IL) [127, 128], and sodium hydroxide/urea [129]. Cellulose derivatives such as cellulose acetate can also be dissolved in various solvents including acetone [130–133], acetone/DMAc [134, 135], and acetone/*N*,*N*-dimethylformamide (DMF) [136–138] to produce ES-CNFs. Chitin is often dissolved in 1,1,1,3,3,3-hexafluoro-2-propanol [116, 139–141] and IL [142, 143] prior to electrospinning. Chitosan—an *N*-deacetylated derivative of chitin—can be electrospun by dissolving it in trifluoroacetic acid (TFA) [117, 144] or aqueous acetic acid solution [145]. With regard to protein nanofibers, degummed silkworm silk or spider silk can be electrospun using 1,1,1,3,3,3-hexafluoro-2-propanol [146–148] and formic acid (FA) [118, 149–152] as solvents. Various types of collagen obtained from calf skin and chicken sternal cartilage are usually dissolved in 1,1,1,3,3,3-hexafluoro-2-propanol [153–158], whereas 2,2,2-trifluorothanol (TFE) [159, 160], FA [161], acetic acid [162], and FA/acetic acid systems [163] are used to dissolve gelatin derived from porcine and bovine skin before electrospinning.

To produce continuous nanofibers of uniform width, the concentration of the biopolymer solution should be carefully controlled in an approached range. The structures of the nanofibers are critically determined by the applied voltage, the injection speed of the syringe pump, the spinning distance, the relative humidity, and the ambient temperature [112–114]. Generally, electrospun BPNFs are long and continuous. They have smooth surfaces and form randomly oriented nonwoven structures. The properties of nanofibers are mainly determined by the molecular structures of the biopolymers and their assembly within the nanofibers during electrospinning. Novel active components are introduced during or after electrospinning to manipulate the structures and performances of the nanofibers. Specifically, CNCs have been integrated with other polymers as building blocks for the fabrication of electrospun nanofibers [164, 165]. As with other electrospun nanofibers [112–114], BPNFs can be formed into many complex structures—such as nanoparticle-coated nanofibers (Figure 4(g)) [166], core-sheath structures (Figure 4(h)) [167], aligned nanofiber structures (Figure 4(i)) [168], ordered arrays, and hierarchical structures—by careful integration with active components during or after electrospinning and by the manipulation of nanofiber alignment, stacking, and folding.

## 3. Nanogenerator Development

Owing to their intrinsic structures and performances, BPNFs are often selected as sustainable building blocks for NG development, and because BPNFs are so diverse, many types of NGs have been constructed. BPNFs are either used directly or chemically modified to form films, mats, membranes, or aerogels for various NGs. They can also be integrated with active materials during or after the formation of the bulk materials. The structure, porosity, roughness, and constituents of the bulk material can be manipulated to further optimize the performance of the NGs. In recent years, BPNF-derived TENGs and PENGs have attracted increasing attention, and BPNFs have also been used to fabricate NGs for the generation of electricity from moisture and osmosis. We will discuss recent advances in the utilization of BPNFs for various types of NGs in the coming sections.

### 3.1. Polysaccharide Nanofiber-Derived NGs

Polysaccharide nanofibers can be produced using various strategies. Therefore, the structures and performances of polysaccharide nanofiber-derived NGs vary considerably. NFC and NFCh can be used directly or modified or integrated with active materials to produce films, mats, membranes, or aerogels for NGs. BC pellicles can be used directly or disintegrated into small pieces to integrate with active components to produce films or aerogels. Because CNC and ChNC are relatively short, they are generally integrated with other polymers or coatings on a substrate before the NGs are assembled. Electrospun polysaccharide nanofibers are usually formed into mats for NGs. The diversity of polysaccharide nanofibers enables various strategies including chemical modification, hybridization with active materials, and tailoring of the structures of bulk materials, for exploiting the advantages of polysaccharide nanofibers and improving the output properties of the as-assembled NGs.

#### 3.1.1. NFC/NFCh-Derived NGs

**Figure 5:**
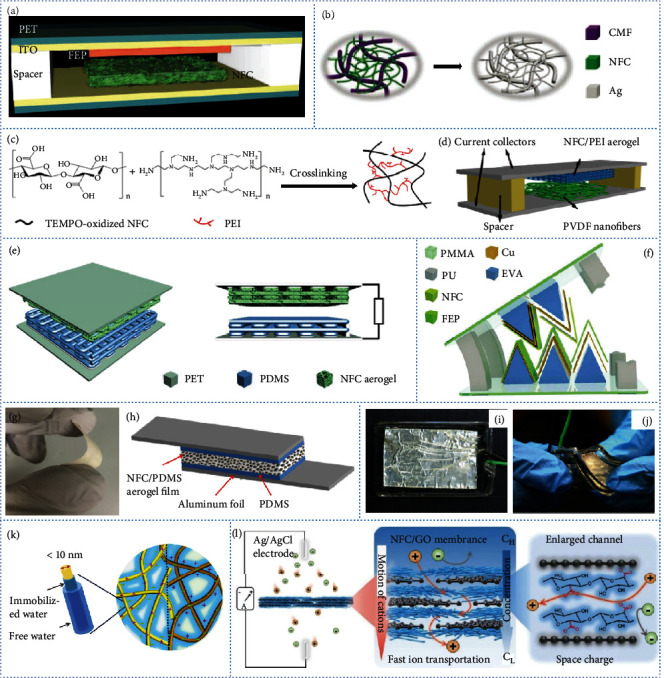
Development of NFC/NFCh-derived nanogenerators (NGs). (a) Schematic diagram showing the assembly of a TEMPO-oxidized NFC film-derived TENG. (b) Schematic diagram showing the construction of an NFC/cellulose microfiber (CMF)/silver composite for a TENG. Schematic illustration of (c) the reaction mechanism of TEMPO-oxidized NFC and polyethylenimine (PEI) and (d) the assembly of a TEMPO-oxidized NFC/PEI aerogel film-derived TENG. (e) Schematic of an all-printed 3D NFC aerogel-derived TENG. (f) Schematic of a gear-like NFC-derived TENG. (g) Digital image of a flexible NFC/polydimethylsiloxane (PDMS) aerogel film. (h) Schematic of an NFC/PDMS aerogel film-derived PENG. Digital images of (i) an NFCh film-derived PENG and (j) the PENG undergoing a flexibility test. (k) Schematic illustration of the voltage induced by the directional movement and neutralization of free ions within hydrated charged nanochannels of an NFC aerogel. (l) Schematic illustration of an NFC/graphene oxide (GO) membrane-derived osmotic energy harvesting system. (a) is reproduced with permission from Ref. [52], copyright 2016 *Nano Energy*. (b) is reproduced with permission from Ref. [179], copyright 2018 *Advanced Functional Materials*. (c) and (d) are reproduced with permission from Ref. [180], copyright 2018 *Nano Energy*. (e) is reproduced with permission from Ref. [181], copyright 2019 *Nano Energy*. (f) is reproduced with permission from Ref. [177], copyright 2019 *Nano Energy*. (g) and (h) are reproduced with permission from Ref. [54], copyright 2016 *Nano Energy*. (i) and (j) are reproduced with permission from Ref. [183], copyright 2018 *Journal of Materials Chemistry A*. (k) is reproduced with permission from Ref. [55], copyright 2020 *Nano Energy*. (l) is reproduced with permission from Ref. [185], copyright 2020 *Materials Horizons*.

Owing to their high aspect ratios and advantageous mechanical properties, NFC and NFCh can be used to fabricate strong and flexible bulk materials for various NGs [169–172]. TEMPO-oxidized NFC was used to prepare a transparent, flexible, triboelectrically positive film. A TENG with an electrical output comparable to that of a typical synthetic polymer TENG device was successfully assembled by pairing the film with FEP (Figure 5(a)) [52]. NFC can also be used as a supporting matrix to integrate and protect active materials such as phosphorene to produce a hybrid film as the active layer of a TENG [173]. Moreover, NFC can be hybridized with active materials and plays multiple roles in the as-assembled TENG. Using vacuum filtration followed by hot pressing or annealing, a flexible and conductive bilayer NFC/silver nanowire film was produced [174]. In a TENG assembled by pairing the two films in parallel, the NFC and silver nanowire layers not only act as the triboelectric and countertriboelectric layers but also as the substrate and electrode, respectively. NFC can also be hybrid with active materials to assemble a TENG that is able to work in a single-electrode mode. A hybrid of NFC and MXene produces a composite dispersion that can be used as a flexible liquid electrode [175]. NFC is used as a dispersant and interlocking agent to promote the interconnection of two-dimensional (2D) MXene nanosheets. An NFC/MXene liquid electrode-based TENG with an open-circuit voltage up to 300 V was produced, which can produce electrical output under various extreme deformations.

Owing to its native chemical composition and structure, cellulose has weak tribopolarity in the triboelectric series. The surfaces of cellulose nanofibers comprise numerous hydroxyl groups. These groups can be regarded as suitable sites for chemical modification or integration with active materials to improve the output performances of TENGs. Integration with carboxyl or nitro groups increases the electron-withdrawing capability of cellulose nanofibers, which therefore readily acquire negative charge. In contrast, the introduction of methyl or amino groups enhances the electron-donating performance of cellulose nanofibers, which are consequently likely to be positively charged [24]. Tribopositive and tribonegative NFC with positive and negative surface charge densities of 62.5 *μ*C m^−2^ and 85.8 *μ*C m^−2^ were prepared by the attachment of methyl and nitro groups, respectively [176]. A TENG assembly comprising paired tribopositive and tribonegative NFC films produces an average voltage output of 8 V and a current output of 9 *μ*A. Silver nanoparticle-coated NFC/polyethylenimine (PEI) films can be fabricated by crosslinking the NFC and PEI by glutaraldehyde activation and then coating the films with nanoparticles [177]. The nanoscale surface and the increase in positive triboelectric polarity enhance the triboelectric output of the TENG.

To increase the porosity and interior surface area and create a rougher surface, three-dimensional (3D) NFC aerogels were exploited by the lyophilization method to form porous triboelectric materials that are useful in TENGs. The output performance of the NFC aerogel-derived TENG is improved by increasing the contact area and electrostatic induction of the porous structure, resulting in the generation of additional charge on the porous surface [178]. The performance of the NFC aerogel TENG can be further improved by integration with active materials using various strategies. The integration of NFC with cellulose microfibers and silver produces a hierarchical nanostructure composite with antibacterial activity for TENG (Figure 5(b)) [179]. Hybridization of NFC with highly tribopositive materials—such as silica fibers, human hair, and rabbit fur—enhances the triboelectric output of the TENG [22]. Introducing novel chemical groups with high electron donating or sucking functionality is an efficient way of improving triboelectric output. The tribopositive polarity of an NFC aerogel can be enhanced by introducing amino groups to improve its electron-donating ability by crosslinking the NFC with PEI (Figures 5(c) and 5(d)) [180] or subjecting the NFC to silanization using aminosilane [178].

TENG performance can be further enhanced by designing and patterning the structures of NFC-based bulk materials because NFC is easily processed. NFC has been explored as a substrate or building block for the construction of intrinsic structures by printing. The voltage output of an all-printed NFC aerogel-based TENG with a 3D hierarchical structure is nearly 175% of that of a TENG assembled by the molding method (Figure 5(e)) [181]. A gear-like TENG can be fabricated by integrating an NFC-based composite with an EVA substrate, which is cut into a triangular columnar body and attached as a tooth-shaped structure (Figure 5(f)) [177]; this 3D flexible space structure increases the frictional contact area. A TENG assembled from three pairs of such gear-like structures has a maximum open-circuit voltage of 286 V.

NFC and NFCh are sustainable piezoelectric materials and are used to fabricate PENGs. The NFC aerogel film was coated with PDMS (Figure 5(g)) [54], which was subsequently inserted between two PDMS films and two aluminum foils to produce a flexible PENG (Figure 5(h)) [54]. The PENG produced a stable output signal under periodic mechanical deformation. The electricity generated by the PENG could directly power 19 blue light-emitting diodes (LEDs) and charge a capacitor by up to 3.7 V. A high-strength TEMPO-oxidized NFC/molybdenum disulfide nanosheet composite film with a longitudinal piezoelectric constant of 31 pC/N was produced [182]. The composite film-based PENG had a maximum output voltage of 4.1 V and a short-circuit current of 0.2 *μ*A. NFCh has been used directly or integrated with PVDF to produce films for PENGs (Figures 5(i) and 5(j)) [183]. NFCh film-based PENGs have an open-circuit output voltage of 22 V and a short-circuit current of 0.12 *μ*A. The incorporation of NFCh into PVDF results in the nucleation of *β* polymorphs in the composite film, leading to an output voltage of 49 V and a short-circuit current of 1.9 *μ*A.

In addition to their utilization in TENGs and PENGs, NFC and NFCh have been used to construct NGs for harvesting electricity from moisture and osmosis. Negatively and positively charged NFC and positively charged NFCh have been used as building blocks to fabricate aerogels with layered structures and oriented pores [184]. With the hydrophilicity and charged states, the aerogels can absorb moisture and generate hydrated nanochannels. Open-circuit voltage was obtained due to the streaming potential formed by a dynamic balance of water absorption and evaporation. Asymmetric ionic aerogels can be constructed from bilayer structures with oriented microscale pores using oppositely charged NFC building blocks (Figure 5(k)) [55]. When moisture is absorbed, the dissociation and diffusion of ions within the hydrated nanochannels induce the directional movement of charge, producing an open-circuit potential of ~115 mV with a maximum short-circuit current of 45 nA. To harvest osmotic energy, TEMPO-oxidized NFC has been integrated with graphene oxide (GO) nanosheets to form a composite membrane (Figure 5(l)) [185]. The introduction of TEMPO-oxidized NFC enlarges the channel, decreases the energy barrier for ion transport, and provides space charge between the pristine GO nanosheets to maintain the ion selectivity. A power density of 4.19 W m^−2^ was achieved by mimicking sea/river water conditions. Besides, a power density of 7.20 W m^−2^ was obtained at 323 K.

#### 3.1.2. BC-Derived NGs

**Figure 6:**
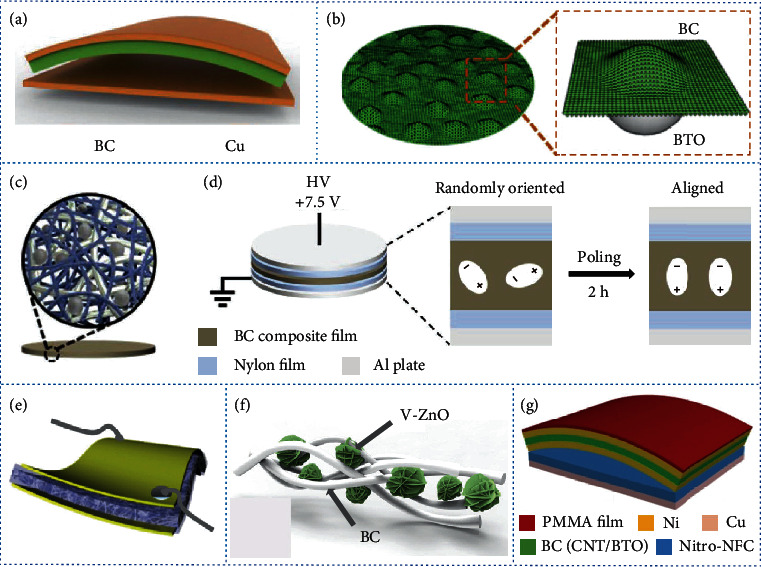
Development of BC-derived NGs. (a) Schematic of a regenerated BC film-derived TENG. (b) Schematic showing the construction of a BC/BaTiO_3_ (BTO) composite film for a TENG. Schematics of (c) the structure of a BC/BTO/silver nanowire composite film and (d) the poling process of a BC/BTO/silver nanowire film-derived TENG. (e) Schematic of a BC/BTO composite film-derived PENG. (f) Schematic showing the construction of a BC/V-ZnO composite film for a PENG. (g) Schematic of a BC/BTO/multiwalled carbon nanotube (CNT) composite film-derived hybrid triboelectric/piezoelectric NG. (a) is reproduced with permission from Ref. [186], copyright 2017 *Nano Energy*. (b) is reproduced with permission from Ref. [187], copyright 2019 *Nano Energy*. (c) and (d) are reproduced with permission from Ref. [188], copyright 2019 *Advanced Functional Materials*. (e) is reproduced with permission from Ref. [189], copyright 2016 *Advanced Science*. (f) is reproduced with permission from Ref. [53], copyright 2018 *Nano Energy*. (g) is reproduced with permission from Ref. [190], copyright 2016 *Nano Research*.

BC is commercially available as a biopolymer nanofiber that can be purchased directly and is therefore widely accepted by researchers as a raw material for NGs. Generally, BC is disintegrated by various mechanical treatments and then regenerated or hybridized with active materials to produce films or aerogels for NGs. A flexible and transparent regenerated BC film was integrated with copper foil to assemble an arch-shaped TENG (Figure 6(a)) [186] that had an accumulative charge of ~8.1 *μ*C m^−2^ and a peak power density of ~4.8 mW m^−2^, at a load resistance of 1 M*Ω* when a force of 16.8 N was applied. To improve the dielectric constant and construct a micro-nanostructure with a rough surface, the disintegrated BC was integrated with dielectric BTO particles to form a composite film (Figure 6(b)) [187], which was assembled with PDMS. The as-obtained TENG had a maximum short-circuit current of 21 *μ*A, an open-circuit voltage of 181 V, and a transfer charge quantity of 76.7 nC. The disintegrated BC was also hybridized with BTO nanoparticles and silver nanowire for use as a conductive ferroelectric composite film (Figure 6(c)) [188]. The as-produced film was utilized as a positive triboelectric layer and a bottom electrode. The electron-donating capability of the film was improved by the poling process in a forward direction (Figure 6(d)) [188]. The as-assembled TENG produced an output voltage of ~170 V and a current of ~9.8 *μ*A under a compressive force of 5 kgf. The optimized output power density of the TENG achieved ~180 *μ*W cm^−2^.

BC can also be used as a flexible polymer matrix for integration with piezoelectric materials to produce PENGs. A disintegrated BC was combined with piezoelectric BTO nanoparticles to produce a composite film. The as-assembled PENG (Figure 6(e)) [189] had an open-circuit voltage of 14 V and a short-circuit current density of 190 nA cm^−2^. The maximum power density was 0.64 *μ*W cm^−2^. Because BC is thin and long and the BC networks were densely permeated with BTO nanoparticles, the PENG was flexible and was able to produce a peak voltage of 1.5 V. Vanadium-doped ZnO (V-ZnO) microflowers were uniformly assembled in the BC film using an in situ synthesis method (Figure 6(f)) [53]. A composite film containing ferroelectric V-ZnO was prerequisite for poling with a high external voltage to enhance output performance. The as-fabricated flexible PENG had an output voltage of 1.5 V, a current density of 80 nA cm^−2^, and a power density of 60 nW cm^−2^.

Hybrid triboelectric-piezoelectric NGs are fabricated by integrating BC and NFC-based materials. A hybrid tribo/piezoelectric NG was developed using a nitro-NFC film as the triboelectric layer and a BC/BTO/multiwalled carbon nanotube composite film as the piezoelectric layer (Figure 6(g)) [190]. The TENG had an open-circuit voltage of 37 V and a short-circuit current density of 1.23 *μ*A cm^−2^, and the PENG had an open-circuit voltage of 22 V and a short-circuit current density of 220 nA cm^−2^. The outputs of the hybrid NG reached 18 V and 1.6 *μ*A cm^−2^, when a full-wave bridge diode was used to integrate the two outputs.

#### 3.1.3. CNC-Derived NGs

**Figure 7:**
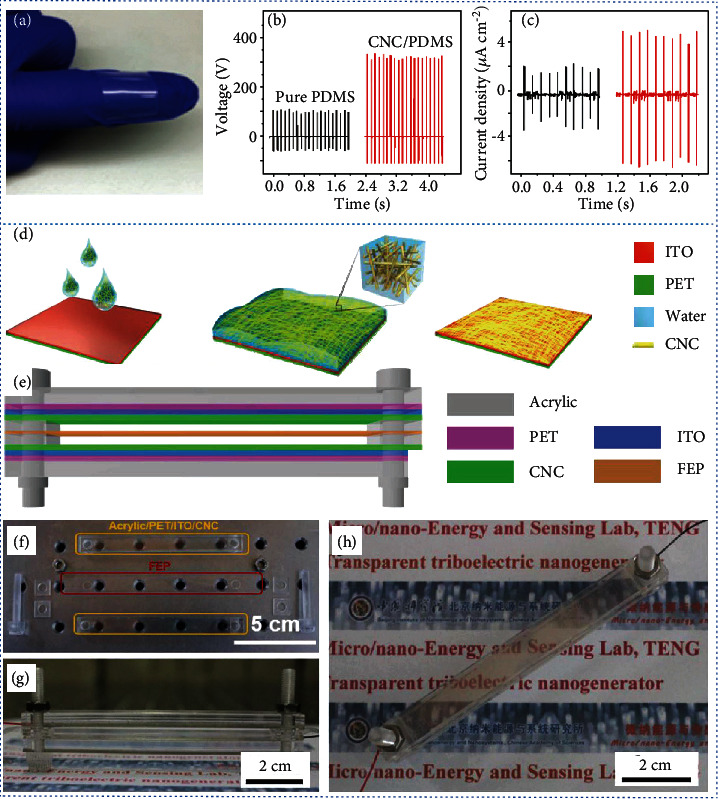
Development of CNC-derived NGs. (a) Digital image of a CNC/PDMS composite film. (b) Open-circuit voltage and (c) short-circuit current density of a pure PDMS film-derived TENG and a CNC/PDMS composite film-derived TENG. (d) Schematic showing the preparation of CNC/indium tin oxide (ITO) electrodes. (e) Schematic of the fabricated CNC-derived TENG. Digital images of (f) the various components, (g) the cross-sectional view, and (h) the top view of the CNC-derived transparent TENG. (a)–(c) are reproduced with permission from Ref. [191], copyright 2017 *Nanoscale*. (d)–(h) are reproduced with permission from Ref. [192], copyright 2018 *Nano Energy*.

CNC can be used to fabricate composites or transparent materials for NGs. CNC flakes were used as dielectric fillers by directionally embedding them in PDMS to prepare a composite film (Figure 7(a)) [191]. The as-assembled TENG had an open-circuit voltage of ∼350 V and a short-circuit current density of ∼5 *μ*A cm^−2^. It exhibited a 10-fold power increase compared to a pure PDMS-derived TENG, under the same periodic compression (Figures 7(b) and 7(c)) [191]. Wood-derived CNC as a triboelectric positive material was deposited on an indium tin oxide (ITO) electrode to form a transparent friction layer/electrode assembly architecture (Figure 7(d)) [192]. A transparent wind-driven TENG was fabricated by assembling two CNC/ITO films and a FEP vibration film (Figures 7(e)–7(h)) [192]. The TENG was capable of generating 2 mW of power, an output voltage of up to 130 V, and a current of 15 *μ*A at a wind speed of 20 m s^−1^.

#### 3.1.4. ES-CNF-Derived TENGs

ES-CNFs can be used as porous nanofiber mats for TENGs. Owing to their intrinsic biodegradability, chemical stability, ready processability, and positive triboelectric polarity, electrospun cellulose acetate nanofibers, which have a high specific surface area, were used as a positive friction layer in TENGs [193]. The ES-CNFs were distributed uniformly with an average diameter of 418 nm. When integrated with a composite nanofiber membrane, the as-assembled TENG with multilayered nanofibers produced plentiful triboelectric charges and can enhance the transfer rate and storage depth of the triboelectric charges. Such TENGs can deliver a power density of 0.13 W m^−2^ with an effective area of 9 cm^2^ at a load of 30 M*Ω*.

### 3.2. Protein Nanofiber-Derived NGs

Research into protein nanofiber-based NGs lags behind the rapid advances in polysaccharide nanofiber-derived NGs. However, protein nanofibers, especially those produced from silk fibers, have gained significant attention in recent years owing to their ease of fabrication, abundance, sustainability, biocompatibility, biodegradability, and advantageous mechanical properties [30]. Because protein nanofibers are diverse, the structures and properties of nanofiber-derived bulk materials differ markedly, resulting in discrepancies in the performances of NGs. Although collagen- and gelatin-derived nanofibers can be used to fabricate NGs, current research on protein nanofiber-based NGs mainly focusses on NFS- and ES-SNF-derived NGs. In the subsequent sections, we will discuss recent advances in the use of NFS and ES-SNFs for various NGs.

**Figure 8:**
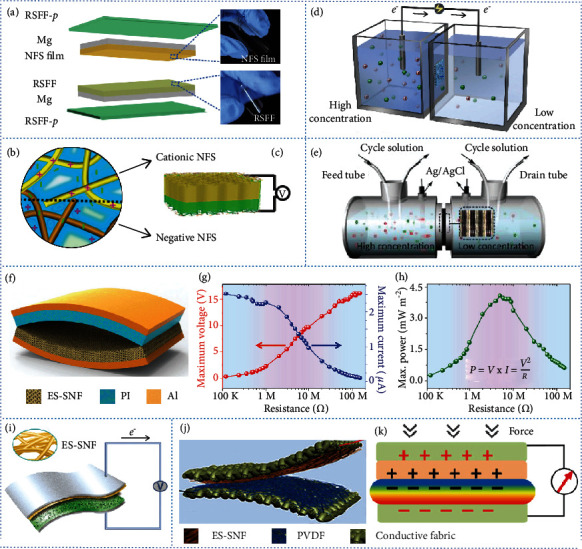
Development of silk nanofiber-derived NGs. (a) Schematic showing the construction of an NFS-derived TENG. Schematics of (b) a bilayer ionic aerogel comprising oppositely charged NFS and (c) a bilayer ionic aerogel-derived NG. (d) Schematic of an NFS membrane-derived osmotic energy harvesting device. (e) Schematic of a GO/NFS/GO membrane-derived osmotic power generator. (f) Schematic of an ES-SNF-derived TENG. The (g) voltage output and (h) power output on the external load resistance of the ES-SNF-derived TENG. (i) Schematic of an ES-SNF and electrospun PVA/MXene nanofiber-derived TENG. Schematics of (j) the construction and (k) the positive interaction effect of an ES-SNF and electrospun polyvinylidene fluoride (PVDF) nanofiber-derived hybrid triboelectric/piezoelectric NG. (a) is reproduced with permission from Ref. [96], copyright 2020 *Nano Energy*. (b) and (c) are reproduced with permission from Ref. [194], copyright 2020 *ACS Nano.* (d) is reproduced with permission from Ref. [56], copyright 2019 *Nano Communications*. (e) is reproduced with permission from Ref. [195], copyright 2020 *ACS Nano*. (f)–(h) are reproduced with permission from Ref. [51], copyright 2016 *Advanced Energy Materials*. (i) is reproduced with permission from Ref. [196], copyright 2019 *Nano Energy*. (j) and (k) are reproduced with permission from Ref. [197], copyright 2018 *Nano Energy*.

NFS can be used directly to fabricate bulk materials for the friction layers of TENGs. A 0.38 nm thick film was produced by integrating ribbon-like NFS with a regenerative silk fibroin film and magnesium to construct an all-silk bio-TENG (Figure 8(a)) [96]. The raw materials used in the TENG were biodegradable and biocompatible. The differences between the microstructures and work functions of the NFS film and the regenerative silk fibroin film are beneficial for increasing the output performance of the TENG. The TENG displayed a maximum voltage of 41.6 V, a current of 0.5 *μ*A, and a power density of 86.7 mW m^−2^.

NFS has also been used to fabricate NGs for the generation of electricity from moisture. Positively charged cationic NFS (∼4 nm thick) have been integrated with negatively charged NFS to form asymmetric ionic aerogels (Figures 8(b) and 8(c)) [194]. When faced with moist air, hydrated and oppositely charged NFS serve as nanochannels for ion transportation and generate ion gradients. Such aerogels can create an optimal open-circuit voltage of up to 121 mV in humid ambient air.

For osmotic energy harvesting, an NFS membrane playing as a screening layer that dominates the ion transport was integrated with an anodic aluminum oxide substrate membrane. An as-generated nanofluidic membrane with asymmetric geometry and charge polarity illustrated a maximum power density of 2.86 W m^−2^, when mixing the artificial seawater and river water at basic conditions (Figure 8(d)) [56]. To further improve the power density, NFS was integrated with GO to construct a multilayer GO-NFS-GO nacre-mimetic membrane as an osmotic power generator (Figure 8(e)) [195]. The NFS mainly served as a nanoscale lock to prevent the free slip of the GO sheets. The intrinsic structures of composite membranes and the synergistic effects of the building blocks enhance interfacial bonding and endow the membranes with long-term stability in saline. The 2D nanofluidic channel configuration decreases resistance to ion transport and offers plenty of storage spaces for ions. An as-generated NG comprising such an NFS-based membrane produced an output power density of up to 5.07 W m^−2^ when mixing seawater and river water.

ES-SNFs have attracted attention as candidates for the fabrication of porous nanofiber membranes. ES-SNF membranes have rougher surfaces than cast silk films. ES-SNFs with diameters of 100–200 nm were paired with a PI film to assemble a TENG (Figure 8(f)) [51], which had a triboelectric surface charge density of up to 1.86 *μ*C m^−2^ and an instantaneous electric power of 4.3 mW m^−2^ at 5 M*Ω* (Figures 8(g) and 8(h)) [51]. The ES-SNFs were also paired with other electrospun nanofibers to construct an all-fiber TENG. Generally, ES-SNF films are used as electron donor layers. When paired with an MXene nanosheet and poly(vinyl alcohol) electrospun nanofibers, an as-fabricated all-electrospun nanofiber TENG exhibits an instantaneous maximum peak power density of 1087.6 mW m^−2^, at a load resistance of 5 M*Ω* (Figure 8(i)) [196]. Owing to its all-fiber structure, the TENG is flexible and foldable and illustrates structure stability under repeated mechanical deformation. All-fiber hybrid triboelectric-piezoelectric nanogenerators have been created by integrating ES-SNFs with electrospun piezoelectric nanofibers. The hybrid NGs were generated by electrospinning silk fibroin and PVDF nanofibers on conductive fabrics [197]. A cloth-shaped device (Figures 8(j) and 8(k)) [197], which had mechanical flexibility and desirable wearing comfort, was obtained by pairing the two components. The voltage, short-circuit current, and power density of the hybrid NG reached 500 V, 12 *μ*A, and 0.31 mW cm^−2^, respectively.

## 4. Conclusion and Outlook

Recent extensive research into BPNFs has demonstrated their considerable potential for use in NGs. BPNFs are derived from abundant and renewable sources and inherit most of the advantages of biological materials, such as advantageous mechanical properties, biocompatibility, and biodegradability. The distinctive structures and properties of BPNFs make them useful for a variety of NGs. BPNF-derived TENGs and PENGs are largely employed. Moreover, BPNFs have also been used in the development of NGs for generating electricity from moisture and osmosis.

In the present review, we have discussed how to obtain nanofibers from biopolymers such as polysaccharides and proteins and how to utilize them to produce a variety of NGs. Various strategies—including exfoliation from biological materials, biosynthesis, and electrospinning—have been developed to fabricate BPNFs with diverse structures and properties. The unique 1D nanofiber structures and physical/chemical functionalities of BPNFs enable the production of as-fabricated bulk materials with exceptional performance improvements for NGs. With regard to the fabrication of NG devices, BPNFs can be used in the following ways: (1) direct use, chemical modification, or integration with active materials to produce films, mats, membranes, or aerogels for NGs; (2) fabricating flexible, foldable, and transparent bulk materials for the development of functional NGs; (3) constructing bulk materials with tailored characteristics including pore size, pore structure, roughness, nanofiber alignment and patterning, nanofiber/matrix composite structure, and complex architecture for optimal NG performance; and (4) fabricating numerous bulk materials for NG development using diverse types of BPNFs as building blocks.

Tremendous effort has been devoted to the assembly of BPNF-derived NGs for various applications. However, the further optimization of the performances and functionalities of NGs faces certain challenges. These include the following: (1) the mass production of BPNFs using simple, cost-effective, and green routes, to provide abundant building blocks for NGs; (2) the development of more effective ways to chemically modify the active groups of BPNFs by introducing more functional groups to modulate their performance; (3) integrating BPNFs with active components and controlling their surface/interface interactions; (4) tailoring the hierarchical structures of bulk materials from the viewpoint of multiple scales to optimize the architecture and output performance of the NGs; (5) fabricating more types of BPNFs and BPNF-derived bulk materials for various types of NGs; (6) focusing greater attention on the structural similarities and differences among the various types of BPNFs; (7) focusing greater attention on the structural and performance advantages of BPNFs and BPNF-derived bulk materials during NG assembly and application; and (8) further designing the chemistry/structure of BPNF-derived materials to create more newly emerging NGs. An exciting future lies ahead for BPNF-derived NGs once these critical issues have been fully addressed.
